# 肺癌手术日间化管理中国专家共识（2024年版）

**DOI:** 10.3779/j.issn.1009-3419.2024.102.24

**Published:** 2024-06-20

**Authors:** 国家卫生健康委医院管理研究所肺癌手术日间化管理专家共识组

**Keywords:** 肺肿瘤, 外科, 日间手术, 医院管理, 专家共识, Lung neoplasms, Surgery, Day surgery, Hospital management, Expert consensus

## Abstract

为高效利用有限的手术资源以缓解肺癌手术的医疗压力、促进国家分级诊疗政策的进一步落实，急需构建肺癌日间手术分级诊疗体系。肺癌日间手术各节点的关键质控有助于提升医疗质量安全与医疗服务效率，促进我国日间手术保持安全有序的良性发展。肺癌手术日间化管理中国专家共识小组凝聚了国内相关领域专家，结合国内外相关专业最新研究成果，基于肺癌日间手术全周期管理视角与患者全生命周期管理视角制定《肺癌手术日间化管理中国专家共识（2024年版）》。该共识主要从术前评估、麻醉管理、手术操作、术后随访、医院-社区一体化构建和应急管理等方面形成共识性意见，旨在为我国肺癌日间手术的开展提供研究支撑和临床指导。

## Abstract (en)

To alleviate the medical burden of lung cancer surgery and facilitate the implementation of the national hierarchical diagnosis and treatment policy, it is imperative to establish a hierarchical diagnosis and treatment system for day surgery of lung cancer. Identifying key quality control checkpoints in day surgery of lung cancer is essential to enhance medical quality, ensure safety, and improve the efficiency of medical services. These efforts aim to uphold a safe and well-structured progression of day surgery practices in China. The Chinese Expert Consensus Group on Day Surgery Management of Lung Cancer has convened national experts in relevant fields and integrated the latest research findings from both domestic and international sources to craft the Chinese Expert Consensus on Day Surgery Management of Lung Cancer (2024 Edition). This consensus is founded on the principles of holistic management of lung cancer surgery and comprehensive patient care throughout their medical journey. It encompasses preoperative assessments, anesthesia protocols, surgical procedures, postoperative care, hospital-community collaboration initiatives, and emergency response strategies. The primary objective of this expert consensus is to furnish research assistance and clinical recommendations to advance the practice of day surgery for lung cancer patients in China.

随着人们健康意识的提高和低剂量螺旋计算机断层扫描（low dose spiral computed tomography, LDCT）在体检与临床中的广泛应用，大量需要手术的肺结节被检出，而肺癌手术的医疗需求与现有医疗资源的矛盾也日益突出^[1]^。日间手术这种高效的择期手术管理模式恰好能够有效地解决当下所面临的“手术资源紧俏”“术前等待时间长”“上下转诊机制不清”等医疗管理难点^[2]^。2022年11月国家卫生健康委员会印发的《医疗机构日间医疗质量管理暂行规定》（以下简称《规定》）要求医疗机构在保障医疗质量安全的前提下，日间医疗为患者提供住院全流程诊疗服务不超过24 h^[3]^。肺癌日间手术相比同样的专科住院手术具有术前等待时间短、恢复快、费用低和患者满意度高等优点，患者、医院和国家三方均可从中受益^[4]^。

外科微创技术（以胸腔镜手术为代表）和加速康复外科（enhanced recovery after surgery, ERAS）理论的临床应用、发展与普及，使微创肺癌手术日间化管理成为可能^[5]^。日间手术不仅可以明显缩短患者术前等待时间、减少住院时间、降低住院费用以及其他交通、住宿费等机会成本，更重要的是还能减轻患者的心理负担^[6]^。此外，肺癌日间手术在提升医疗服务效率、减少手术资源浪费的同时，还构建了肺癌加速康复外科体系与肺癌日间手术分级诊疗体系，响应了我国分级诊疗政策，极大地促进了肺癌日间手术在我国的规范化发展^[7]^。因此，开展肺癌日间手术是一种有益的尝试，有望为更多肺癌患者提供更加优质便捷的医疗服务。

肺癌根治性切除手术属于四级手术，肺癌日间手术的开展对医疗机构级别、设施设备、手术医生、行政管理和患者自身等都有较高的要求，无论国内医疗机构开展的日间手术是中心式还是分散式，亦或是独立的日间手术中心，其管理须达到同质化，以保证医疗质量与患者安全^[8]^。本共识推荐意见共10条，覆盖肺癌日间手术全流程，适用于我国二级以上医疗机构或专科疾病防治机构。

**共识1：医疗机构开展肺癌日间手术应提前做好顶层设计，包括组织与运行管理、平台构建与多学科团队建设。**

肺癌日间手术与传统住院肺癌手术的最大差异在于医疗服务模式与流程的改变，医疗质量本质并无差异，对医疗设施设备的要求标准并未因此削弱，对手术医生的要求反而更高。因此，已成熟开展肺癌手术的医疗机构在条件与时机成熟的情况下可考虑肺癌日间手术，但在组织与运行管理、平台构建、多学科团队建设等医疗质量控制方面建议严格管控，具体如下。

**组织与运行管理**

根据《规定》要求设置日间医疗质量管理委员会，制定明确的管理制度，包括患者评估制度、随访制度、医务人员培训制度等。加强院科两级责任制日间医疗质量管理，动态管理日间手术医生的授权与技术目录，定期对日间肺癌手术进行医疗质量监测、分析、预警和反馈等^[3]^。

**平台构建**

建议医疗机构根据实际情况开展中心式（日间病房统一管理）与分散式（病房在各个科室）肺癌日间手术，麻醉和手术室相关设备与住院手术同质化^[4]^。中心式日间手术对于医疗机构要求相对较高，须建造日间手术中心或在原病房基础上改造成集中收治日间手术患者的医疗单元，建议病房、护士站、手术室、收费室、等待区域等相对集中于同一楼层或同一栋楼，一方面缩短术后患者返回病房途中缺乏密切监护的时间，降低患者术后返回病房途中的风险；其次减少患者在院内各科室往返的时间，提升患者住院体验和满意度。分散式肺癌日间手术则直接在专科住院部指定床位或区域收治，而医疗管理流程不同于其他住院患者，适用于尝试开展肺癌日间手术的初期探索阶段，便于节省资源并快速开展。

肺癌手术对手术室的要求为十万级，根据《综合医院建筑设计规范》JGJ49-88、《医院洁净手术部建筑设计规范》GB50333-2002等相关规范要求建设设计^[9,10]^，严格划分为限制区、半限制区和非限制区，避免交叉感染。设计包括洁净走廊、洁净库房、麻醉准备、刷手间、污物走道、清洗间、值班室、治疗室、处置室、护士站、医生休息室、男女更衣室等^[11]^。

病房建议设计集中收治的日间手术病房与手术室在同一楼层，减少术后患者返回病房途中缺乏密切监护的时间。病房的大小与床位数根据开展的单位实际情况而定，可根据不同的手术类型设计房间类型，总体原则是便于医护团队观察处理、保障患者舒适隐私。每张观察床位配备完善的床头设施，应设有中央供氧、中央吸引、中央呼叫对讲和床头照明系统，病房内各类监护仪器（心电监护仪、血压检测仪、血氧饱和度检测仪等）、抢救的配置须与住院专科病房一致，这也是日间手术病房所必须满足的条件。除了对于硬件设施的要求之外，也可根据实际条件和需求增设财务室方便患者结算，设置儿童病房或家庭式病房缓解患者的焦虑情绪等^[12]^。分散式则与专科住院病房共用同一病区资源与医疗设施，建议专科住院病房指定床位或区域收治肺癌日间手术患者，便于医疗管理。

**多学科团队建设**

肺癌日间手术团队由外科医生、麻醉医生、护士、康复科医生、管理者和营养师共同组成，共同讨论后形成“可操作、可评估、可重复”的围手术期方案（管道管理、疼痛管理、饮食管理创新等）和肺康复方案^[13⇓-15]^，如管道管理包括使用18 F尿管替代传统胸腔闭式引流管，并不予安置尿管，在减轻患者疼痛、提升舒适度的同时促进患者早期下床活动，并有利于患者术后加速康复^[16⇓-18]^；个性化疼痛管理采用预防性镇痛和多模式镇痛方案，术中加用肋间神经阻滞麻醉，可显著改善患者术后疼痛症状^[19]^；饮食管理围手术期通过中链甘油三酯（medium chain triglyceride, MCT）饮食促进患者术后胃肠功能快速恢复并缩短术后住院时间^[20]^。各科室和医护人员既有明确分工又有密切合作，充分发挥团队的作用。基于多学科团队诊疗模式可使患者肺部相关并发症发生率、术后住院时间、住院费用显著降低，在保证医疗质量的基础上，极大地提升患者满意度^[21]^。

**共识2: 医疗机构开展肺癌日间手术应严格审核与授权手术医生，并进行动态管理。建立基于医疗、术式与患者三个维度的关键医疗质量控制标准，保障患者安全与医疗同质化。**

医疗质量控制既是开展肺癌日间手术的基础，也是发展的前提。从日间手术服务流程来看可分为入院前、住院中和出院后，影响肺癌日间手术的关键要素分为患者维度、医生维度和术式维度，因此需要综合以上要素制定严格的患者准入标准、动态管理手术医生授权以及严格筛选肺癌手术方式。具体建议如下。

**手术医生准入**

建议肺癌日间手术医生要求具有独立胸腔镜手术资格，且有200例以上独立胸腔镜手术经验，通过胸外科专科业务主任的推荐，并获得医院日间手术对应的术式授权^[8]^。

**术式筛选**

肺癌日间手术需要根据病灶大小、位置、分期等情况综合考虑，建议选择肺实质外1/3、肺结节直径<3 cm的患者，且肺叶切除可以达到治疗目的^[22]^。手术方式为肺叶/肺段/楔形切除和淋巴结采样/清扫，复杂、创伤大和出血风险高的肺癌手术禁止进入日间流程，如支气管成形术、全肺切除术、扩大肺癌切除术等。其次，不同肺叶的多发结节、肿瘤导致困难肺门、门钉淋巴结、肿瘤距离隆突<2 cm、肺部结节考虑为转移瘤等均不适合日间手术。肺癌患者伴发严重疾病、手术复杂、麻醉时间超过3 h、中转开胸或出血>800 mL的患者，建议转专科住院处理。

**患者准入**

建议肺癌日间手术患者应全部满足以下条件：（1）年龄≤70岁^[23]^；（2）吸烟指数<400年支，并且术前戒烟至少4周以上^[24,25]^；（3）第一秒用力呼气容积（forced expiratory volume in one second, FEV_1_）≥1.5 L，术后FEV_1_%（postoperative FEV_1_%, ppoFEV_1_%）和术后肺一氧化碳弥散量百分比（postoperative diffusing capacity of the lungs for carbon monoxide percentage, ppoDLCO%）≥60%^[26,27]^；（4）无哮喘、气道高反应，无慢性阻塞性肺疾病（chronic obstructive pulmonary disease, COPD）、急性上呼吸道感染、阻塞性睡眠呼吸暂停综合征等呼吸系统疾病^[28,29]^；（5）合并高血压者术前血压控制在160/100 mmHg以下；60岁以下同时合并糖尿病的患者血压应控制在140/90 mmHg以下；（6）无心肌梗死病史，无心力衰竭、心律失常、冠心病、冠脉支架置入等心脏疾病；（7）糖尿病患者术前糖化血红蛋白（hemoglobin A1c, HbA1c）<8.5%，空腹血糖≤7.8 mmol/L，随机或餐后2 h血糖<10 mmol/L^[30,31]^；（8）美国麻醉医师协会（American Society of Anesthesiologists, ASA）分级≤2级^[32]^。以下情况不推荐行日间手术：（1）身体质量指数（body mass index, BMI）>28 kg/m^2^；（2）月经期或者有出凝血功能障碍；（3）明显肝肾功能异常；（4）神经系统疾病控制不稳定者；（5）多发肺结节考虑肺内转移或有远处转移；（6）放化疗、靶向治疗后；（7）肺结核、胸膜炎病史，或怀疑胸腔重度粘连；（8）肺气肿、肺大疱明显，术后延长肺漏气风险较大者；（9）既往胸部手术史；（10）阻塞性睡眠呼吸暂停综合征。

日间手术不仅仅需要患者自愿参与，建议还需要满足以下社会因素：（1）患者陪护人员身体状况良好、有责任心和良好的沟通能力，住院期间能全程陪护，出院后能胜任照护工作；（2）患者住所距离医院不超过1 h车程，便于紧急情况时能及时就医，若患者出院后选择社区医院继续康复治疗则可不严格限制患者住所距离；（3）患者需要保持联系畅通，确保医院可以定期随访，医务人员可以根据患者术后症状提供康复指导和建议，并评估患者是否发生需要医疗干预的并发症^[33]^；（4）异地患者医保不支持术前门诊检查报销，患者需要根据自身情况考虑是否选择日间手术。

**共识3：医疗机构开展肺癌日间手术术前准备要求同专科住院患者，推荐肺癌日间手术时须严格评估患者身心状态与拟行手术方式等因素，患者完成相关术前检查后须通过麻醉医生术前评估与手术医生的再次评估。**

在《规定》中组织与运行管理明确要求建立机构日间医疗患者、病种、技术的遴选机制和医务人员的审核授权管理机制，并组织实施。开展肺癌日间手术术式与手术医生授权须通过医院日间管理委员会授权，并制定统一的临床路径，以确保肺癌日间手术患者医疗质量的同质化管理。患者将术前检查前移至门诊，肺癌日间手术患者须通过医院授权的日间手术专科医生门诊评估，在门诊初步评估符合条件的患者，并且征得患者及家属同意后开具术前检查和入院证，确定预手术日期。待患者完善术前检查并通过麻醉医生与手术医生的再次评估后，方能确定最终手术日期，具体流程见图1。术前检查与传统住院手术应一致，包括血常规、肝肾功能、电解质、术前凝血常规、输血前全套、血型；肺功能；心电图；头、胸、上腹部相关影像学检查。多发结节或者既往肿瘤史患者必要时可完善正电子发射计算机断层显像（positron emission tomography/CT, PET/CT）检查，有合并疾病患者根据术前评估情况增加检查项目，如甲状腺功能减退症患者须复查甲状腺激素，心电图异常患者必要时加做心脏超声等^[34]^。建议医疗机构可根据当地医保报销政策制定术前检查纳入报销的最长时限，2周内完成术前检查，以保证检查结果的时效性和准确性。患者需要根据自身经济情况、医疗保险支付范围等方面综合考虑是否选择日间手术。如有异常结果，专科医生需要进一步检查评估，以明确患者是否能进入日间手术流程。只有ASA分级≤2级的患者才能进行日间手术下一步的预约；若ASA分级为3级，须麻醉医生与主刀医生同时评估确认后才能预约，根据病情安排收入专科住院病房进行手术治疗。确认预约排程后进行日间手术术前注意事项提示、健康宣教和康复训练。术前1 d预约护士会再次与患者确认手术日期、术前准备等相关信息^[35]^。

**图1:**
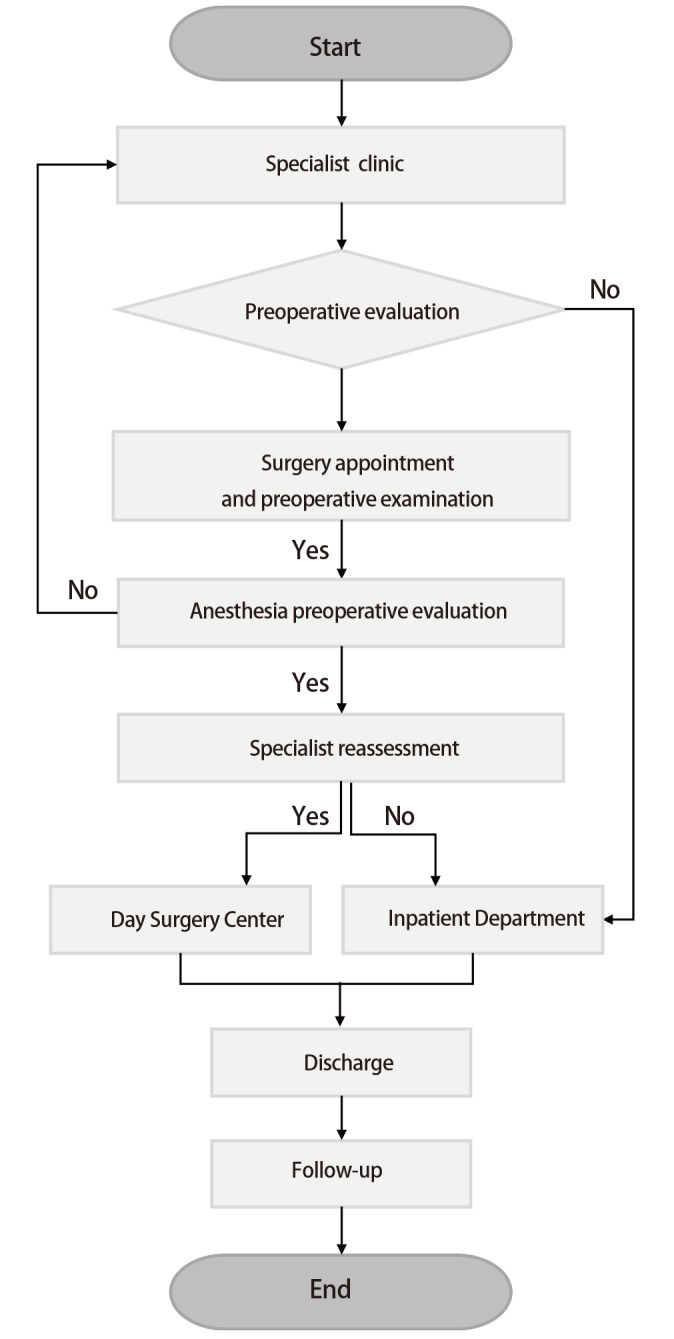
肺癌日间手术患者预约入院流程

**共识4：肺癌日间手术方式微创和开放均适用；微创手术（包括机器人手术）切口可选择单孔、两孔和三孔；切除范围包括肺叶/肺段（同侧手术小于3段）/楔形。**

手术方式要确保手术过程顺利，尽可能以简单易行的操作完成手术，手术时间在3 h内。手术方案中操作流程应以缩短手术时间为主要考虑因素，并合理控制麻醉深度和术中补液量，减少出血。而常规手术器械包中多数器械使用率极低，通过精简手术器械包可明显缩短清点器械时间和不必要器械的安装与拆卸等操作，并且不影响手术效率和出血量，优化器械后可进一步缩短手术时间^[36]^。手术操作应轻柔，避免过多的牵拉、钳夹、翻动肺组织，以减少对肺组织、支气管的损伤。术中操作应避免过度分离肺裂和肺实质，优先处理肺门再处理肺裂可减少肺漏气^[37]^，切割肺裂和段间平面推荐使用切割缝合器，避免使用能量器械直接烧灼分离。松解肺韧带可减少术后残腔，手术结束后应仔细检查是否存在漏气情况，必要时使用Prolene线或者防漏气生物材料修补漏气处。

手术方法选择微创手术（包括机器人手术），建议开胸手术尽量不纳入日间手术，若术中中转开胸术后建议转入专科病房进一步治疗。手术切除范围包括肺叶/肺段（同侧手术小于3段）/楔形。手术可采用三孔、单操作孔、单孔或者机器人系统，主刀医生可根据自身手术经验选择熟悉流畅的手术方式，日间手术应避免实施双侧肺结节同期切除或全肺切除术。肺叶切除推荐“单向式胸腔镜肺叶切除”^[38]^，对于肿瘤直径<2 cm、实性成分占比（consolidation tumor ratio, CTR）<0.25的肺结节，根据病灶位置合理选择楔形切除^[39]^，以减少解剖段门的创面和手术时间，降低肺漏气和肺复张不良的发生率。总之，在保证手术安全的前提下，尽可能以短平快的理念优化手术操作和流程。尽可能减少复杂肺段切除，肺叶切除和楔形切除患者术后恢复相对更为满意。

**共识5：肺癌日间手术麻醉管理遵循快通道“四早”原则，采用双腔支气管导管插管全身麻醉。围手术期疼痛管理推荐以非甾体类抗炎药（nonsteroidal antiinflammatory drugs, NSAIDs）为基础的预防性镇痛、术中区域神经阻滞为基础的少阿片多模式镇痛。**

**麻醉管理**

肺癌日间手术的麻醉遵循早苏醒、早进食、早下床、早出院的“四早”原则。建议麻醉方式采用双腔支气管导管插管全身麻醉 ，暂不推荐不插管全身麻醉。因为日间肺癌手术不涉及全肺、支气管袖式等，使用支气管双腔插管进行肺隔离时一般情况下均可以选用左侧双腔支气管导管，以降低插管难度，同时有利于获得术侧良好的肺塌陷状态^[40]^。气管插管手法可采用环状软骨推移法，提高左侧支气管导管一次到位率^[41]^。准确的双腔管定位和肺塌陷是保障外科操作的前提，建议借助纤支镜或可视双腔管等设备完成，以减少盲插和位置调整相关的气道损伤。麻醉药物原则上选择作用时间短、起效迅速、消除快、无蓄积的药物。胸腔镜肺癌日间手术需要良好的肌松效果，避免膈肌运动影响手术快速精准操作，应选用短效非去极化肌松药物，术毕注意残余肌松拮抗。围手术期常规应用预防恶性呕吐的药物，如糖皮质激素、5-羟色胺3（5-hydroxytryptamine 3, 5-HT3）受体拮抗剂等药物治疗，术后恶心呕吐高危患者推荐全凭静脉麻醉。此外咀嚼口香糖、内关穴按摩等非药物措施也对减轻术后恶心呕吐有效^[42]^。术中除常规麻醉监测外，必须监测呼气末二氧化碳（end tidal carbon dioxide, ETCO_2_）。脑电双频指数（bispectral index, BIS）等设备监测麻醉深度，有助于控制麻醉用药，加快术后清醒。围手术期推荐使用小潮气量、低平台压，适合呼气末正压（positive end-expiratory pressure, PEEP）策略。单肺通气时，高频低潮气量（4-6 mL/kg）通气可以减少单肺通气肺损伤，同时根据患者个体情况保持PEEP通气压力水平为5-10 cmH_2_O，可使单肺通气达到传统潮气量通气相同的氧合水平^[43]^。术中不留置尿管，推荐液体零平衡、缩短术前禁饮禁食时间、术后早进食的管理策略。

**疼痛管理**

肺癌日间手术的围手术期疼痛管理应确保患者静息状态下无痛、咳嗽或深呼吸状态下轻微疼痛，不影响睡眠和饮食，能自由下床活动，无恶心呕吐、头晕、嗜睡等不良反应，促进患者无痛、舒适、安全地度过围手术期。围手术期建议采用预防性镇痛、多模式镇痛和少阿片化镇痛的理念，预防性镇痛药物选择对乙酰氨基酚和NSAIDs，不推荐使用患者自主镇痛（patient controlled analgesia, PCA）^[19]^，具体如下：（1）术前预防性镇痛：患者进入手术室前2 h静脉使用NSAIDs和/或对乙酰氨基酚；（2）术中镇痛：避免大剂量长效阿片类药物的使用，术毕采用以罗哌卡因为主的镇痛药，进行广泛肋间神经阻滞；（3）术后镇痛：以阿片类药物为主的静脉PCA术后恶性呕吐发生率较高，且不利于出院后阿片类药物管理，不推荐日间手术使用。建议术后镇痛使用对乙酰氨基酚和NSAIDs，可联合口服氨酚羟考酮或芬太尼透皮贴剂等措施^[44]^。

**共识6：尽量减少肺癌日间手术患者围手术管道（静脉管道、各类监护仪器导线、尿管、胸腔引流管），缩短留置时间。**

研究^[45]^表明，胸腔镜肺癌手术时间约2 h，术中输液量约1500 mL ，患者术中尿量约400 mL，而目前胸腔镜肺癌手术大多在1 h左右完成，术中输液量控制在500 mL左右。对于胸腔镜肺癌手术患者，术中无论安置尿管与否，术后因尿潴留重新安置尿管的发生率并无统计学差异（2.6% vs 2.8%, P=0.729）。因此患者术中可以常规不安置尿管，也不需要术后留置尿管^[46,47]^，这样不仅提高了患者的舒适度和恢复速度，有利于患者术后早期下床活动，也能节约医疗费用、减少护理工作量。建议肺癌日间手术预估手术时间<3 h、术中输液量<2000 mL时可不安置尿管。

传统的胸腔引流方式是术后疼痛和伤口延迟愈合的主要原因，不仅会延长住院时间，还降低了患者的住院舒适度，建议肺癌日间手术使用周长<20 F的硅胶引流管，留置1根。患者返回病房3-4 h后，鼓励下床活动或坐位休息，指导辅助患者咳嗽，并完成胸片检查。患者咳嗽无明显漏气，胸片无明显积气、积液、肺不张等，引流液性质无明显异常时，考虑术后6-10 h拔除引流管。咳嗽有轻微漏气、肺复张欠佳时需延迟拔管，必要时应用负压吸引，手术次日晨基本可顺利拔管。如持续漏气或者持续引流鲜血性积液，则需转入普通病房继续治疗。

**共识7：肺癌日间手术患者围手术期饮食原则为按需、低脂饮食，建议选择MCT饮食方案。**

营养管理通过优化围手术期的处理措施减少代谢应激反应，如糖代谢紊乱、胰岛素抵抗、肠道菌群紊乱等，以达到减少并发症、促进患者快速康复、缩短住院时间的目的。患者术前6 h禁食固体饮食，建议术前2 h前饮用含12.5%碳水化合物的清流质250 mL，术后2 h口服温开水100 mL，无明显不适可继续口服开胃流质250 mL（含有机酸和电解质）。术后4-6 h口服低脂型肠内营养粉50 g，术后第1天开始正常进食，术后第1-3天推荐MCT饮食方案^[48]^。

营养支持贯穿于围手术期的各个阶段，包括术前营养评估、术前常规进行肠道准备、术前缩短禁食时间、术前口服清流质进行代谢准备、术后早期快速康复饮食等^[49]^。

**共识8：肺癌日间手术患者出院前使用麻醉后离院评分表（postanesthesia discharge score, PADS）严格评估患者是否达到出院标准。专科医生确定患者出院后去向，转专科治疗或下转社区医院、康复医院等医疗机构进一步观察。**

为保障患者围手术期的质量和安全，必须制定标准化的出院标准，需要医生与护士在不同时间节点共同评估，医疗评估需要胸外科专科医生完成。肺癌日间手术患者出院标准与专科病房大致相同，须满足：（1）胸腔引流管可在出院前顺利拔除；（2）无明显皮下气肿（范围未及对侧胸壁）；（3）术后胸片无明显积气积液（<20%）；（4）无明显胸闷气短，不依赖氧气；（5）体温<38 ^o^C。建议胸外科专科医生和日间病房医生使用麻醉术后通用的PADS评分表进行评估^[50]^。PADS评分包括血压和脉搏、活动能力、术后恶心呕吐、出血和疼痛，每项评分分别为0、1、2分，患者总评分≥9分方可准予出院。若患者评分≤8分或有口服药物不能控制的疼痛、过敏反应、呼吸困难等，则需要胸外科医生评估后予以相应的处理。对于预计不能在24 h内出院的患者，建议根据实际病情转入专科病房继续治疗，或下转社区医院、康复医院等医疗机构进一步观察和康复治疗。

**共识9：肺癌日间手术患者住院期间突发事件应急处理预案与专科住院患者相同，如术中发生出血等事件，则术后直接转入专科病房。出院后增设并保证日间手术急诊绿色通道和医院-社区一体化提供延续性医疗服务的通畅性，入院后纳入病房管理模式。**

建议医疗机构开展肺癌日间手术之初须建立完善的应急预案，包括住院期间和出院后^[51]^。住院期间突发事件应急处理预案与专科住院患者相同，应关注肺癌日间手术相关并发症的应急处置，如气胸、皮下气肿、出血等引起的患者呼吸困难、血压下降等。手术过程中突发事件的应急处理应遵循预防为主、常备不懈的方针。遇到以下情况应立即退出日间手术流程，术后转入专科病房：（1）术中出血>800 mL，或因出血或其他原因中转开胸；（2）术中发现胸膜粘连闭锁或重度粘连，术后可能发生延长漏气或引流渗出较多；（3）术中处理困难肺门或门钉淋巴结，对肺实质解剖损伤较明显；（4）术中出现过敏性休克、恶性心律失常或危急值结果。管床医生应仔细观察、充分评估病情，出现病情变化时积极对症处置，做好生命体征监测，联系主刀医生协调处置，必要时请相应专科会诊协助诊治。经积极治疗后，患者病情平稳，评估达到出院标准后可正常出院，并注意加强患者出院后随访。若评估患者病情不允许24 h内出院，由主刀医生协调安排患者经绿色通道转入专科病房继续观察治疗，做好相关医疗文书记录^[52]^，报告日间手术中心负责人，统筹协调处置。

出院后30 d内需对患者开展至少3次随访，若患者出现相应并发症时，随访人员应指导患者或家属做简单的处理或救治，告知患者到急诊就诊或通知急诊科进行处理。建议医疗机构建立急诊绿色通道，积极联系主刀医生或者专科病房住院总协助处置患者，必要时收入专科住院治疗，并报告日间手术中心负责人，参与协调和沟通，同时做好相应记录。

**共识10：肺癌日间手术患者出院后可至康复门诊，由康复专科医生统一制定康复方案，康复可在社区、康复医院或家庭中进行。**

康复科治疗师指导患者术后早期深呼吸训练和有效咳嗽，可以改善肺不张、预防肺部感染。鼓励患者早期下床活动，能够改善患者氧转运的能力，维持并提高肌肉的肌力和耐力以及肌肉的柔韧性，维持正常的神经系统功能，减少焦虑和压力^[53]^。术后康复科床旁指导患者气道廓清技术、肢体活动、呼吸练习等，并制定居家康复的运动和呼吸训练方案，以促使患者尽早恢复，尽快参与日常生活。

按计划出院的患者可居家康复，应尽量实施医院-社区双重管理，患者术后伤口拆线换药、饮食、睡眠及康复训练等常规处理可下转社区管理。若患者出现并发症、社区医务人员不能判断患者病情或病情加重社区处理条件不够时，应及时与医院随访人员联系处理，甚至联系主刀医生指导处理，决定患者到医院门诊或急诊就诊。专科评估患者是否需要入院，需再手术时启用绿色通道，进一步保障日间手术患者围手术期的医疗质量与安全。

随着日间手术管理逐渐成为我国择期手术管理的主流趋势，对手术效率和安全性的重视也日益增强。肺癌手术日间化管理是胸外科手术日间化管理的首要探索领域。除了肺结节/肺癌的胸腔镜日间手术外，胸腔镜下纵隔肿瘤切除、手汗症等手术也逐渐纳入日间化管理范畴。目前，我国许多医疗机构也在积极开展相关工作。本次共识汇集了四川大学华西医院在肺癌手术治疗、肺康复和日间手术管理等方面的前期研究成果，整合国内相关领域的专家意见，为胸外科日间手术提供经验借鉴和参考的同时，旨在推动我国分级诊疗政策的进一步落实，为患者提供公平、安全和高质量的医疗服务。
