# 更正声明

**DOI:** 10.3779/j.issn.1009-3419.2024.103.01

**Published:** 2024-07-20

本刊2024年第27卷第6期刊登的题为《肺癌手术日间化管理中国专家共识（2024年版）》[国家卫生健康委医院管理研究所肺癌手术日间化管理专家共识组. 肺癌手术日间化管理中国专家共识（2024年版）. 中国肺癌杂志, 2024, 27(6): 405-414.]一文中，第414页编写组成员中“屠正良”更正为“屠政良”。特此更正。

Erratum: Chinese expert consensus on day surgery management of lung cancer (2024 edition).

Consensus Group of Experts on Day Surgery Management of Lung Cancer, Hospital Management Research Institute of the National Health Commission.

Zhongguo Fei Ai Za Zhi, 2024, 27(6): 405-414.

In the version of this article initially published, the error appeared on page 414. In the consensus writing team, “屠正良” should be “屠政良”.

